# Bioinformatics Applications to Reveal Molecular Mechanisms of Gene Expression Regulation in Model Organisms

**DOI:** 10.3390/ijms222111973

**Published:** 2021-11-05

**Authors:** Yuriy L. Orlov, Tatiana V. Tatarinova, Anastasia A. Anashkina

**Affiliations:** 1 The Digital Health Institute, I.M. Sechenov First Moscow State Medical University of the Ministry of Health of the Russian Federation (Sechenov University), 119991 Moscow, Russia; nastya@eimb.ru; 2 Agrarian and Technological Institute, Peoples’ Friendship University of Russia, 117198 Moscow, Russia; 3 Life Sciences Department, Novosibirsk State University, 630090 Novosibirsk, Russia; 4 Natural Science Division, La Verne University, La Verne, CA 91750, USA; ttatarinova@laverne.edu; 5 Engelhardt Institute of Molecular Biology, Russian Academy of Sciences, 119991 Moscow, Russia

Gene expression regulation at the transcriptome, genome, cell, and tissue levels is a complex phenomenon demanding the development of bioinformatics tools. Molecular mechanisms of human disease progression as well as gene expression in laboratory animal models are being studied using sequencing approaches coupled with advanced analytics. Modern computational approaches depend on the ability to reconstruct gene networks and model the protein structure. This Special Issue “Molecular Mechanisms of Gene Expression: Bioinformatics of Gene Regulations and Structure” continues the collection of papers published in this journal in the wake of a series of bioinformatics conferences held in Russia in 2020 (https://bgrssb.icgbio.ru/2020/). The presented analytic techniques were discussed at the BGRS-2020 (Bioinformatics of Genome Regulation and Structure/Systems Biology) biannual computational biology meeting in Novosibirsk, highlighting recent advances in the evolution, biomedicine, and biotechnology areas. This collection continues the studies in the field of bioinformatics of gene expression initially presented in *Frontiers in Genetics* [1], *PeerJ* (https://peerj.com/collections/72-bgrs-sb-2020), and then in *International Journal of Molecular Sciences* [2].

This collection of papers showcases insights into the fields of genomics, transcriptomics, and proteomics, as well as some works conducted in model organisms. The current Special Issue contains eight research manuscripts and one review, each concerning a model or a pipeline applied to extract information useful for understanding the molecular mechanisms of gene expression regulation. We continue the previously published set of paper collections with an overarching theme of bioinformatics initially focused only on biomedical applications (https://www.mdpi.com/journal/ijms/special_issues/Medical_Genetics_Bioinformatics) [2,3]. This Special Issue is focused on the deciphering of the molecular mechanisms underpinning chronic diseases and disorders. 

The current series of post-conference journal Special Issues started with the coverage of the Bioinformatics of Genome Regulation and Structure (BGRS) [4,5,6] conferences and the related Schools on Systems Biology and Bioinformatics (SBB) held in Novosibirsk, Russia [7,8], and it was later completed by other international conferences on genetics, such as the Belyaev Conference—2017 [9] and “Centenary of Human Population Genetics” in 2019 [10,11]. The papers in this issue present novel bioinformatics models on gene expression regulation, alternative splicing, chromatin immunoprecipitation-sequencing (ChIP-seq) technology to analyze transcription factor (TF) binding to DNA, as well as some insights into the molecular mechanisms of human diseases. 

We open this collection of papers with the review by Rivka C. Stone et al. [12] on the genomics of human fibrotic diseases. The authors discussed fibrosing disorders systematically, characterizing the initial acute injury that drives unresolved inflammation, describing the genomics studies, and summarizing various therapies and gene network modeling studies. 

The next series of articles considers TF binding that controls gene expression on a genomic scale. Keunsoo Kang and colleagues [13] analyzed Forkhead box protein M1 (FOXM1) binding sites in human cell lines. FOXM1 is a critical TF that plays an important role in regulating a common set of genes involved in the cell cycle in several different cell types (MCF-7, K562, SK-N-SH, GM12878, and ECC-1). The authors showed that FOXM1 might control the gene set through interaction with the NFY proteins, while cell type-specific genes were predicted to be regulated by enhancers that interact with FOXM1 and cell type-specific TFs.

Flavia Goncalves Fernandes and colleagues [14] studied somatic copy number alterations (CNAs) and associated genes in clear cell renal cell carcinoma (ccRCC) in Brazilian patients who underwent nephrectomy. The study was designed to evaluate the chromosomal profile of CNAs in ccRCC tumors and explore clinical associations. Bioinformatics analysis discovered 19 genes that were mapped to CNA significant regions, including *SETD2*, *BAP1*, *FLT4*, *PTEN*, *FGFR4*, and *NSD1*. This study generated the first CNA landscape among Brazilian patients with ccRCC.

The following articles presented general bioinformatics modeling of gene expression regulation at the transcription level. Victor Levitsky et al. [15] studied the cooperative binding of TFs to genomic DNA as the mechanism of transcription regulation based on ChIP-seq data. The authors applied a novel computer package to predict pairs of spaced or overlapped motifs based on a single ChIP-seq dataset. An extensive analysis of 119 ChIP-seq datasets for 45 human TFs with annotated occurrences of anchor motifs found that for almost all TF families, the co-occurrence of overlap between the motifs of target TFs and more conserved partner motifs was significantly greater than that for less conserved partner motifs. Co-occurrence with an overlap of pairs of less and more conserved motifs of partner TFs could explain why a substantial fraction of ChIP-seq data lacked conserved motifs of target TFs. TF binding recognition based on nucleosome occupancy profiles in eukaryotes using a yeast model show presence o less conserved motifs [16]. Recently developed computer programs [17] could allow for accurate detection of ChIP-seq binding peaks from sequencing data.

Xiaomin Zhang and co-authors [18] characterized the effect of alternative splicing of the sirtuin genes on their gene products’ diversity. Among seven human sirtuin genes (*SIRT1* to *SIRT7*), five sirtuin genes (i.e., *SIRT1, SIRT2, SIRT3, SIRT5*, and *SIRT6*) had more than one isoform, and among them, the *SIRT6* gene had nine different isoforms. Most sirtuin isoforms resulted in a loss of a part of the sequence, such as the nuclear localization signal and mitochondrial targeting signal, or shortened lengths of protein domains, including N- or C-terminus domains or the catalytic domain. The human *SIRT1* gene’s three isoforms had different effects on the mitochondrial oxygen consumption rate. The findings of this study showed that alternative splicing increases sirtuin gene diversity and affects subcellular localization and function, which could enhance the complexity of the gene regulation of mitochondrial respiration, metabolism, and cardiac function in the process of maturation and aging.

Frida Belinky et al. [19] analyzed stop codons within prokaryotic protein-coding genes. The authors demonstrated that in-frame stop codons and read-through events (i.e., the suppression of in-frame stop codons) appear to be common biological phenomena in prokaryotic organisms. The evolutionary conservation of stop codons within protein-coding genes suggests that many of them are not pseudogenes. The authors concluded that in-frame stop codons may be an important mechanism of regulation; such codons might lead to a significant reduction of protein expression levels that could bring about benefits in the adaptation of the organisms. The roles of purifying and positive selection in the evolution of stop codons were discussed in another study [20].

Several articles in this issue presented applications of gene expression analysis in model organisms. The deleted in azoospermia like (*DAZL*) gene plays an important role in germ cells] development and maintenance in chickens. Deivendran Rengaraj et al. [21] detected a set of genes that are likely to have direct associations with *DAZL* by applying various in silico prediction tools. The expression patterns of the *DAZL* gene and its interacting genes were studied using a whole-transcriptome sequencing approach.

Martín-Hernández and colleagues [22] performed a comparative transcriptomic analysis of the body wall tissue between wild and farmed sea cucumber *Isostichopus badionotus.* The transcriptome of the body wall tissue from wild and farmed *I. badionotus* was studied for the first time using an RNA sequencing approach. A set of metabolic pathways that are critical for the effective handling and accretion of nutrients and energy or the clearance of harmful cellular metabolites were either disrupted or dysregulated in farmed organisms. Therefore, wild *I. badionotus* could be considered a suitable alternative to other widely used species, although the potential of farmed *I.* *badionotus* appeared to be unclear.

Ivan Petrushin et al. [23] analyzed cooperative interaction of *Janthinobacterium* sp. SLB01 and *Flavobacterium* sp. SLB02 in the diseased sponge *Lubomirskia baicalensis*. Endemic freshwater sponges that dominate in Lake Baikal, Central Siberia, Russia, are multicellular filter-feeding organisms that represent a complex consortium of a variety of eukaryotic and prokaryotic species. The authors described a possible mechanism of the joint occupation of the ecological niche in the freshwater sponge microbial community. This study broadened our understanding of the symbiotic relationship between microorganisms and freshwater sponges in Lake Baikal [24].

Thus, the current Special Issue on bioinformatics shows that gene expression regulation studies are in high demand [1,2]. The guest editors are happy to announce the Special Issue topic at MDPI *IJMS* on medical genomics (https://www.mdpi.com/journal/ijms/special_issues/Medical_Genetics_2021) and next BGRS-2022 conference in Russia (https://bgrssb.icgbio.ru/2022/). We wish that the readers find these papers to be interesting and stimulating, and we are continuing to collect papers on gene expression regulation based on novel sequencing and computational approaches [25], omics technologies, networks, and pathway analysis [26].
